# Melkersson-Rosenthal Syndrome: A Case Treated With Infliximab

**DOI:** 10.7759/cureus.104378

**Published:** 2026-02-27

**Authors:** Francisca A Correia, Adriana Costa, Claudemira Pinto, Vanessa Chaves, Jorge Almeida

**Affiliations:** 1 Internal Medicine Department, Unidade Local de Saúde de São João, Porto, PRT

**Keywords:** diagnosis, granulomatous cheilitis, infliximab, melkersson-rosenthal syndrome, orofacial swelling, treatment

## Abstract

Melkersson-Rosenthal Syndrome (MRS) is a rare disorder of unknown etiology, clinically characterized by a triad of recurrent orofacial swelling with facial palsy and a fissured tongue. Diagnosis is based on physical findings and history, with a biopsy potentially needed for histological confirmation. We present a case of a 40-year-old woman, referred by her stomatologist to our Department of Internal Medicine for granulomatous cheilitis. After excluding other etiologies and in the presence of a compatible biopsy, the diagnosis of MRS was made. There is no standardized treatment for MRS, and most therapeutic decisions are based on isolated case reports and small case series; management is, therefore, individualized according to patient response. In the current reported case, after several different therapeutic regimens had failed, the patient was treated with infliximab and has been improving progressively thereafter.

## Introduction

Melkersson-Rosenthal syndrome (MRS) is a rare condition marked by recurrent orofacial edema, repeated episodes of facial palsy, and a fissured tongue [1]. The classic clinical triad is very rare; most patients present with only one or two of its components, being classified as monosymptomatic and oligosymptomatic, respectively [2]. Facial soft tissue swelling is the most common symptom of MRS (75-100%), followed by facial nerve palsy (30-90%) and, finally, fissured tongue (30-77%) [1]. Besides the three cardinal symptoms, migraine, headache, and dizziness were also present in half of the patients in one case series [3].

It is important to distinguish granulomatous cheilitis (CG) and orofacial granulomatosis (OFG). CG is a rare disorder characterized by chronic swelling of the lips due to granulomatous inflammation, which was described by Miescher for the first time in 1945 [4,5]. OFG is a term used for the first time in 1985 by Wiesenfield that encompasses both MRS and CG [4,5]. In the published literature, these two terms are used concurrently and often described as the same entity [4,5]. These two entities are clinically and histologically indistinguishable [4,5]. Indeed, it is well understood that both OFG and CG could represent a monosymptomatic form of MRS [4,5].

In 1928, Melkersson described the coincidence of facial palsy with angioneurotic edema for the first time, and Rosenthal added the occurrence of lingua plicata to form the classical triad in 1931 [4]. The prevalence of MRS is estimated at 0.08% in the general population; nonetheless, this figure is likely underestimated because the condition is often misdiagnosed or inadequately diagnosed [2,5].

The onset of symptoms is most commonly between 25 and 40 years, with a female preponderance (2:1), and no racial and/or geographical preference [3,5]. Although the etiology of MRS is not fully understood, viral infections, allergic factors, and genetic influences have been implicated, and the condition is often considered to have an autoimmune basis [5].

MRS is diagnosed through clinical evaluation and patient history, with biopsy reserved for cases in which confirmation is necessary [1]. Histopathologically, this syndrome is characterized by noncaseating epithelioid cell granulomas, lymphedema, fibrosis, vasodilation, and congestion [5].

Currently, there is no specific therapy for MRS. Corticosteroids have historically been considered the mainstay of treatment [3]. However, in some patients, these agents are not enough, and other immunosuppressants are used based on small case series. Tumor necrosis factor-α (TNF-α) inhibitors have been reported to be of benefit in various reports [6].

Herein, we describe a successful case of MRS treated with infliximab after several different therapeutic regimens had failed.

## Case presentation

A 40-year-old woman diagnosed with CG was referred to our Department of Internal Medicine by her stomatologist in 2020. This condition was detected after a lip biopsy was carried out in 2018, which revealed a chronic inflammatory infiltrate with confluent epithelioid granulomas with multinucleated giant cells and no necrosis, compatible with CG. The search for microorganisms was negative.

**Figure 1:**
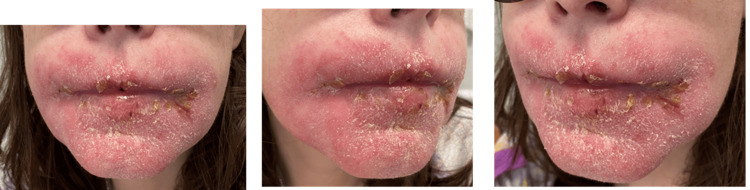
Angular cheilitis lesions and an erythematous and swelling upper lip before treatment.

Personal history included obesity, submitted to gastric bypass in 2007, with subsequent pernicious anemia, with an additional deficiency component (vitamin deficits and menstrual losses). The physical examination revealed a fissured tongue, angular cheilitis lesions, and an erythematous and swollen upper lip (Figure 1).

The patient denied alcohol abuse, recent traveling, blood transfusion, high-risk sexual behaviors, tattoos, intravenous drug use, or consumption of herbal preparations. She also denied any family history or contact with forest areas and animals.

Blood tests showed iron-deficiency anemia, without other changes in blood count or vitamin deficiencies (the patient was under supplementation). There was no renal dysfunction or ionic imbalance, and no evidence of cytocholestasis, hyperbilirubinemia, hypoalbuminemia, or coagulopathy. C-reactive protein, erythrocyte sedimentation rate, thyroid function, and muscle enzymes were normal (Table 1). Urine analysis was normal.

**Table 1:** Blood tests.

Blood tests	Results	Reference range
Hemoglobin (g/dL)	11.2	12-16
White blood cells (x10^9^/L)	10	4-11
Platelets (x10^9^/L)	190	150-400
Transferrin saturation (%)	10	20-50
Ferritin (ng/mL)	95	200-360
Folic acid (ng/mL)	6.6	2.2-17.5
Vitamin B12 (pg/mL)	305	187-883
Urea (mg/dL)	20	10-50
Creatinine (mg/dL)	0.6	0.51-0.95
Sodium (mEq/L)	141	135-147
Potassium (mEq/L)	4.1	3.5-5.1
Chlorides (mEq/L)	107	101-109
C-reactive protein (mg/L)	2.3	<3
Erythrocyte sedimentation rate (mm/1ªh)	11	0-30
Aspartate aminotransferase (U/L)	12	10-31
Alanine aminotransferase (U/L)	15	10-31
Gamma glutamyl transferase (U/L)	8	7-32
Alkaline phosphatase (U/L)	44	30-120
Total bilirubin (mg/dL)	0.6	<1.2
Direct bilirubin (mg/dL)	0.03	<0.4
Albumin (g/L)	40	38-51
Prothrombin time (seconds)	12	11-13.5
Activated partial thromboplastin time (seconds)	26	21-35
Myoglobin (ng/mL)	29	<146.9
Creatine kinase (U/L)	15	10-149
Thyroid-stimulating hormone (μUI/mL)	0.6	0.35-4.94

Angiotensin-converting enzyme (ACE) levels were normal, and serology for human immunodeficiency virus, hepatitis B, hepatitis C, and syphilis was negative. The Mantoux test measured 2 mm, and the interferon-gamma release assay (IGRA) was negative (Table 2).

**Table 2:** Serological tests.

Serological tests	Results	Reference range
Human immunodeficiency virus	Negative	-
Hepatitis B virus	Negative	-
Hepatitis C virus	Negative	-
Syphilis serology	Negative	-
Mantoux test (mm)	2	<5
Interferon gamma release assay	Negative	-

Protein electrophoresis, serum immunoglobulin A (IgA), immunoglobulin M (IgM), and immunoglobulin G (IgG) levels were all normal; antinuclear antibody (ANA) and antibodies against extractable nuclear antigens (anti-ENA) were negative; there was no complement consumption; antineutrophil cytoplasmic antibodies against myeloperoxidase (ANCA-MPO) and anti-double-stranded antibody (anti-dsDNA) were positive at low titer (25 UI/mL and 75 UI/mL, respectively), but when repeated became negative; human leukocyte antigen B (HLA-B) 51/52 was positive (Table 3). 

**Table 3:** Autoimmunity blood tests.

Autoimmunity blood tests	Results	References range
Angiotensin-converting enzyme (U/L)	30	20-70
Protein electrophoresis		
Total proteins (g/L)	70	64-83
Albumin (%)	53.2	49.7-64.4
Alpha globulin 1 (%)	7.2	4.8-10.1
Alpha globulin 2 (%)	11.3	8.5-15.1
Beta globulin 1 (%)	9.4	7.8-13.1
Beta globulin 2 (%)	9.3	7.8-13.1
Gamma globulin (%)	17.6	10.5-19.5
Alpha/beta ratio	1.6	0.99-1.81
Immunoglobulin A (mg/dL)	90	78-312
Immunoglobulin M (mg/dL)	65	55-300
Immunoglobulin G (mg/dL)	1300	650-1500
Antinuclear antibody	Negative	-
Antibodies against extractable nuclear antigens	Negative	-
C3c (mg/dL)	100	83-177
C4 (mg/dL)	26	12-36
Antineutrophil cytoplasmic antibodies against myeloperoxidase (UI/mL)	25	<20
Anti-double-stranded antibody (UI/mL)	75	<70
Human leukocyte antigen B 51/52	Positive	-

The patient underwent upper endoscopy and colonoscopy, a facial computed tomography (CT) scan, and CT enterography, with no relevant findings. A chest X-ray was also performed, without significant changes.

A lip lesion biopsy revealed epithelial acanthosis and focal parakeratosis, areas with dermis edema, capillary dilatation, and noncaseating granulomatous inflammation, compatible with MRS. Histologically, the search for microorganisms was also negative (*Mycobacterium tuberculosis *and *Treponema pallidum*).

The patient underwent several treatments without reaching a total resolution of the condition and with several recurrences. For one year (2020), the patient received intermittent corticosteroid therapy during disease flares (maximum (max) dose 20 mg/day), with good response. However, relapse consistently occurred during dose tapering below 10 mg/day.

In early 2021, the patient received intralesional triamcinolone injections (three injections administered monthly), with no clinical improvement. In February 2022, treatment with hydroxychloroquine (HCQ) was initiated, starting at 200 mg/day and increasing to a maximum of 400 mg/day, with minimal clinical response. Therapy was continued for only two months due to the development of a maculopapular rash on the lower limbs. An attempt to control the rash with antihistamines was unsuccessful.

Later that year, oral methotrexate (MTX) was initiated, with a poor clinical response. Treatment was maintained for only three months, starting at 7.5 mg/week and increasing to a maximum of 10 mg/week. Dose escalation to 12.5 mg/week was not tolerated due to gastrointestinal symptoms (nausea and vomiting) and pancytopenia, including anemia (minimum (min) 7g/dL), neutropenia (min 0.6 x 10^9^/L), and thrombocytopenia (min 90 x 10^9^/L).

**Figure 2:**
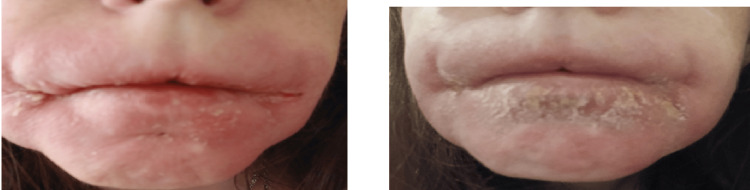
Angular cheilitis lesions and an erythematous and swelling upper lip after treatment with infliximab.

The following year, the patient was proposed and accepted for biological treatment with infliximab (5 mg/Kg intravenous at weeks zero, two, and six, then every eight weeks), with good tolerance and gradual improvement in the condition (Figure 2). When publishing the article, the patient had already taken four doses without complications.

## Discussion

MRS is a rare disorder classically characterized by a triad of symptoms: OFG, facial palsy, and lingua plicata [5]. However, in most cases, MRS does not present with the complete classical triad, which makes diagnosis challenging. In such situations, the clinical presentation of these features, supported by histopathological evidence of noncaseating granulomatous inflammation, and exclusion of other conditions, confirms the diagnosis of MRS [7,8]. In our case, the patient presented with two components of the classic triad (OFG and lingua plicata), and the etiological workup began with the exclusion of other possible diagnoses.

The occurrence of granulomatous lesions in the orofacial region may also be a component of Crohn’s disease or sarcoidosis, or a result of tissue reaction to foreign bodies, as well as granulomatous infectious diseases such as tuberculosis, making it essential to exclude these diagnoses [7]. The absence of constitutional and/or respiratory symptoms, as well as the lack of imaging abnormalities at the pulmonary level, combined with negative Mantoux and IGRA tests, makes the diagnosis of tuberculosis unlikely. Sarcoidosis was excluded after a negative ACE and a normal chest X-ray. Despite iron-deficiency anemia, Crohn’s disease was also excluded after the absence of radiological findings in CT enterography and endoscopic procedures.

Differential diagnosis of MRS also includes systemic lupus erythematosus (SLE), dermatomyositis, neoplasia, and infections beyond tuberculosis [4]. The autoimmune study revealed some positive results, but at low titers and without meeting the criteria for the diagnosis of autoimmune disease, particularly SLE. Low inflammatory markers, negative viral and syphilis serologies, and the absence of microorganisms on histological examination made an infectious etiology unlikely. In addition, a normal facial CT scan and a biopsy without evidence of malignancy argued against a neoplastic process.

Based on her symptoms, histopathological findings and the exclusion of other related conditions, the diagnosis of MRS was made. A standardized therapy for MRS does not exist; traditionally, corticosteroids have been the mainstay of treatment [3,5]. If this treatment fails, other immunosuppressants can be considered. According to recent literature, several therapeutic options have been proposed for MRS, ranging from immunosuppressive and antimicrobial agents, such as azathioprine, MTX, and HCQ, to biologic therapies, including anti-TNF-α monoclonal antibodies (infliximab and adalimumab), as well as nonsteroidal anti-inflammatory drugs (NSAIDs) [5]. Our patient was initially treated with corticosteroids, followed by other immunosuppressants, such as HCQ and MTX. However, this therapeutic approach failed to achieve sustained clinical improvement.

Given the proposed role of tumor necrosis factor in granuloma formation, anti-TNF-α therapies, including adalimumab and infliximab, have been employed to treat conditions such as psoriasis, Crohn’s disease, and sarcoidosis [3,9]. Some authors report that CG can occur several years before the onset of intestinal involvement in Crohn's disease and accounts for about 0.5% of its parenteral manifestations [1]. On this basis, the literature reports beneficial results in cases of MRS treated with infliximab and adalimumab [9,10]. Taking all this into consideration, the decision to start a biological treatment with infliximab was made, and the patient showed significant improvement. Although more studies and case reports are needed, our case is another example of therapeutic success with infliximab.

## Conclusions

MRS remains a disease with an unknown etiology and is challenging to treat. There is no standardized therapy regimen, and, as a consequence, treatment has to be chosen from various agents suggested in small case series. After several therapeutic schemes in this patient, and due to the evidence of some successful cases described, it was decided to start treatment with a biological agent-infliximab. This case is yet another example of the difficulty in treating this type of patient.
